# Comparative analysis of *HER2* copy number between plasma and tissue samples in gastric cancer using droplet digital PCR

**DOI:** 10.1038/s41598-020-60897-4

**Published:** 2020-03-06

**Authors:** Boram Kim, Soo Kyung Nam, Soo Hyun Seo, Kyoung Un Park, Sang-Hoon Ahn, Do Joong Park, Hyung-Ho Kim, Woo Ho Kim, Hye Seung Lee

**Affiliations:** 1 Department of Laboratory Medicine, Seoul National University College of Medicine, Seoul, 03080 Republic of Korea; 2 Department of Laboratory Medicine, Seoul National University Hospital, Seoul, 03080 Republic of Korea; 3 Department of Pathology, Seoul National University Bundang Hospital, Seongnam, 13620 Republic of Korea; 4 Department of Laboratory Medicine, Seoul National University Bundang Hospital, Seongnam, 13620 Republic of Korea; 5 Department of Surgery, Seoul National University College of Medicine, Seoul, 03080 Republic of Korea; 6 Department of Surgery, Seoul National University Bundang Hospital, Seongnam, 13620 Republic of Korea; 7 Department of Pathology, Seoul National University College of Medicine, Seoul, 03080 Republic of Korea

**Keywords:** Gastric cancer, Diagnostic markers

## Abstract

In this study, we measured the human epidermal growth factor receptor 2 (*HER2*) copy number in both tissue and plasma samples of gastric cancer patients by using droplet digital polymerase chain reaction (ddPCR) method. Eighty gastric cancer patients were enrolled and both formalin-fixed and paraffin-embedded tissue and preoperative plasma samples were collected. HER2 status was determined by HER2 immunohistochemistry (IHC)/silver *in situ* hybridization (SISH) in tissue samples and ddPCR of the target gene *HER2* and the reference gene eukaryotic translation initiation factor 2C, 1 in both tissue and plasma. The concordance rate of tissue HER2 status determined by IHC/SISH and *HER2* ddPCR was 90.0% (72/80), and the sensitivity and specificity of tissue ddPCR were 85.0% and 95.0%, respectively. The concordance rate of plasma ddPCR and IHC/SISH was 63.8% (51/80). The sensitivity, specificity, positive predictive value, and negative predictive value of plasma *HER2* ddPCR were 37.5%, 90.0%, 79.0%, and 59.0%, respectively. As *HER2* measurement by tissue ddPCR showed a high concordance rate with HER2 status by IHC/SISH, it could replace tissue IHC/SISH testing in gastric cancer. These findings may contribute to the development of tissue and plasma HER2 testing that would be useful in daily practice.

## Introduction

Gastric cancer is the fifth most common cancer worldwide, and second in Asia^1^. In 9% to 38% of gastric cancers, the human epidermal growth factor receptor 2 (*HER2*) gene is amplified or overexpressed^2^. Because *HER2* gene encodes a transmembrane tyrosine kinase receptor that regulates cell proliferation, apoptosis, adhesion, migration, and differentiation, the amplification and overexpression of *HER2* gene result in cancer progression through the abnormal cell signaling pathway^3^.

Trastuzumab is a monoclonal antibody that targets the extracellular domain of HER2 and inhibits the proliferation and survival of tumours^4^. The phase III trastuzumab for Gastric Cancer (ToGA) demonstrated that the chemotherapy and trastuzumab combination therapy has a survival gain compared to chemotherapy alone^5^. The adoption of trastuzumab as a standard targeted therapy agent for HER2-positive gastric cancer has drawn the importance of HER2 testing^6^.

According to the National Comprehensive Cancer Network Clinical Practice Guidelines in Oncology (NCCN guidelines) and the guideline from the College of American Pathologists, American Society for Clinical Pathology, and the American Society of Clinical Oncology, assessment for HER2 overexpression and/or amplification should be performed with the tumour tissues by immunohistochemistry (IHC) and fluorescence or silver *in situ* hybridization (FISH or SISH)^7–9^. However, most patients who need to be tested for HER2 status have inoperable, advanced, or metastatic gastric cancers^10^; therefore, it is difficult to acquire enough tissues for HER2 testing. Moreover, the variations in IHC and FISH/SISH results, especially due to the intratumoural heterogeneity of gastric cancer, make the HER2 testing questionable^11^.

Liquid biopsy, the analysis of tumour material by the sampling of blood or other body fluids, is a new alternative for cancer diagnosis, prediction for prognosis and residual disease, treatment selection, and monitoring of disease burden^12^. In particular, the droplet digital polymerase chain reaction (ddPCR), where a template is divided into each droplet for individual PCR reactions, is considered as one of the best methods suitable for the analysis of circulating cell-free DNA (cfDNA) because of its high sensitivity and accuracy^13^.

In this study, we aimed to investigate tissue and plasma *HER2* copy number (CN) by using ddPCR in gastric cancer patients and evaluate the utility and compatibility of tissue and plasma *HER2* measurement in gastric cancer.

## Results

### HER2 status determined by various testing methods and cutoff values

**Figure 1:**
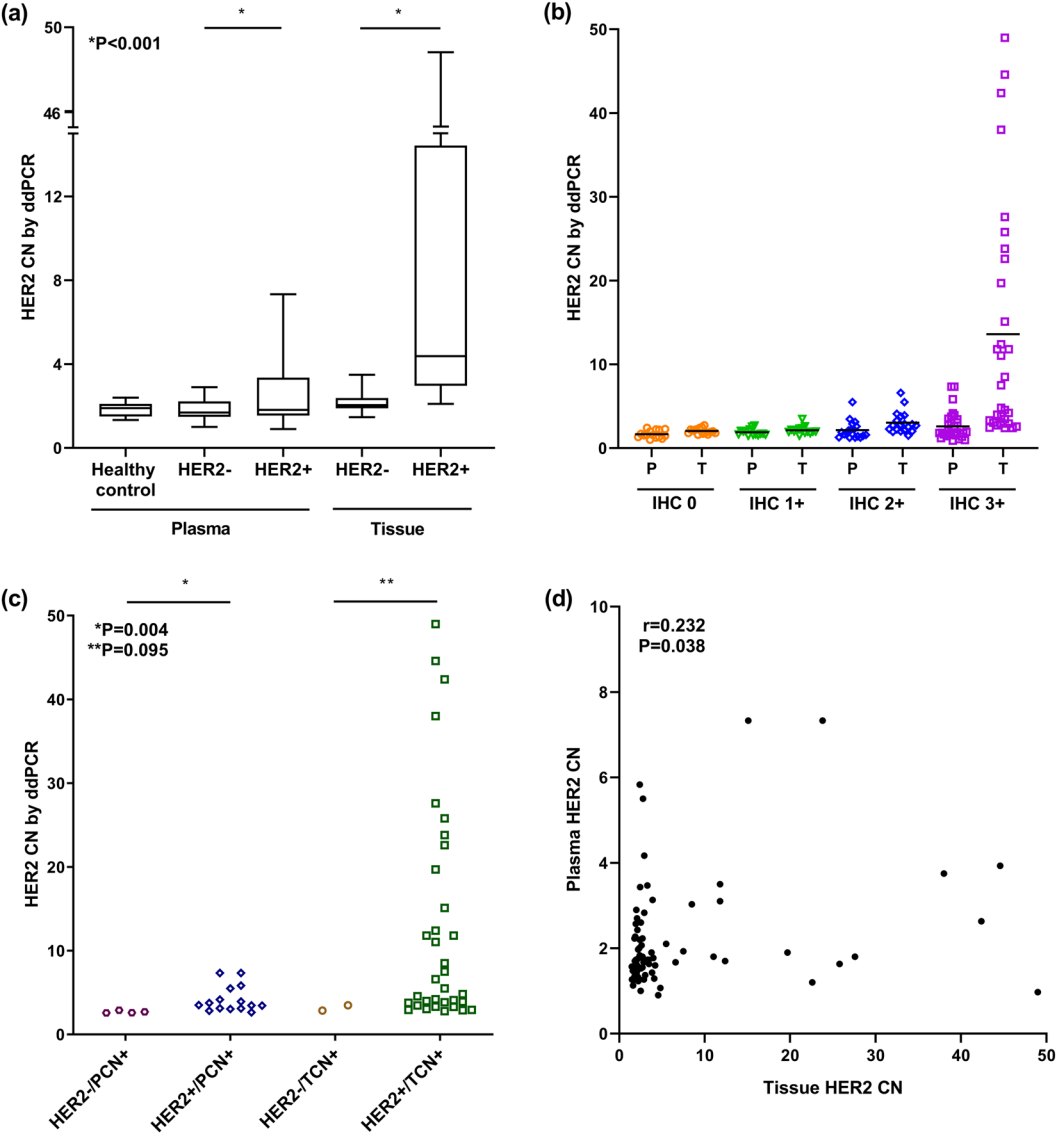
*HER2* copy number measured from tissue and plasma using ddPCR. (**a**) Tissue and plasma *HER2* copy number distribution in healthy control, HER2 negative, and HER2 positive group. (**b**) *HER2* copy number distribution of tissue and plasma according to the HER2 grade. (**c**) *HER2* copy number distribution of tissue and plasma *HER2* positive assessed by ddPCR. (**d**) Correlation of plasma *HER2* copy number with tissue *HER2* copy number. CN, copy number; HER2−, HER2 negative group; HER2+, HER2 positive group; IHC, Immunohistochemistry; P, Plasma; T, Tissue; PCN, Plasma copy number; TCN, Tissue copy number.

Gastric tissues and plasma of 80 patients were tested for *HER2* CN by ddPCR, and HER2 status of each case was determined by IHC and/or SISH analysis in gastric cancer tissues. *HER2* amplification by SISH was observed in 9 out of 19 cases with HER2 IHC 2+; forty cases were HER2-negative and 40 HER2-positive. Considering tissue HER2 status by IHC and/or SISH as the gold standard, tissue *HER2* CN by ddPCR in HER2-positive cases was significantly higher than HER2-negative cases (P < 0.001; Fig. 1a). Plasma *HER2* CN by ddPCR in HER2-positive cases was also significantly higher than HER2-negative cases with statistical significance (P < 0.001; Fig. 1a).

To set a threshold for *HER2* amplification in cell-free plasma samples, we collected 15 healthy volunteers’ blood samples and performed ddPCR of both *HER2* and eukaryotic translation initiation factor 2C, 1 (*EIF2C1*) genes, three times for each sample, except when it was not available. The mean of the plasma *HER2* CN was 1.860, and the standard deviation (SD) 0.325. The cutoff value of *HER2* amplification positivity was defined as the mean plus 2 SDs, and the value was 2.510. As all *HER2* CNs of normal controls were <2.510, it is appropriate to define *HER2* CN ≥ 2.510 as positive *HER2* amplification in cell-free plasma samples.

Meanwhile, we set a threshold for *HER2* CN in tissue samples by ddPCR using receiver operating characteristics (ROC) curve comparing HER2-positive gastric cancer tissue to HER2-negative tissue because the range of *HER2* CN was different between plasma and tissue samples (see Supplementary Fig. S1). The area under the curve (AUC) of *HER2* CN by ddPCR was 0.963 (95% confidence interval, 0.928–0.998) for the diagnosis of *HER2* amplification. The optimal cutoff value was determined as 2.750, and the sensitivity was 85.0% and specificity 95.0%.

### The concordance between various HER2 testing results

We compared *HER2* CN by ddPCR of gastric cancer tissues to the HER2 status by tissue IHC and/or SISH (Table 1). Forty-four patients were *HER2*-negative, and 36 patients were *HER2*-positive, as determined by tissue ddPCR. The overall concordance rate of the two tests was 90.0% (72/80). Tissue *HER2* CN with ddPCR showed an increasing trend according to the HER2 IHC grade (P < 0.001, Fig. 1b).

**Table 1:** Comparison between *HER2* copy number by ddPCR and HER2 status by tissue IHC and/or SISH.

		Tissue IHC and/or SISH		Total
		Negative	Positive	
Tissue ddPCR	Negative	38	6	44
	Positive	2	34	36
Plasma ddPCR	Negative	36	25	61
	Positive	4	15	19
Total		40	40	80

IHC, immunohistochemistry; SISH, silver *in situ* hybridization; ddPCR, droplet digital polymerase chain reaction.

Six patients showed the negative result with tissue *HER2* ddPCR and the positive result for tissue HER2 IHC/SISH. We reviewed hematoxylin and eosin (H&E) stained slides for estimating tumour purity and HER2 IHC slides for the positively stained area (data not shown). Among the six discrepant cases, three showed intratumoural HER2 heterogeneity, and positive area by HER2 IHC was 30%, 10%, and 50%, respectively. The other three cases had low tumour purity (40%, 50%, and 50%, respectively).

In addition, *HER2* amplification of cfDNA was measured with ddPCR and compared with tissue IHC and/or SISH results (Table 1). Sixty-one patients were *HER2*-negative, whereas 19 patients were *HER2*-positive with cfDNA ddPCR. The concordance rate of cfDNA ddPCR and tissue IHC and/or SISH was 63.75% (51/80) with Cohen’s kappa of 0.275. The sensitivity and specificity of plasma *HER2* ddPCR were 37.5% and 90.0%, respectively. The tissue and plasma *HER2* CN by ddPCR were lower in CN-positive/HER2-negative cases than CN-positive/HER2-positive cases (P = 0.095 and P = 0.004, respectively; Fig. 1c).

**Figure 2:**
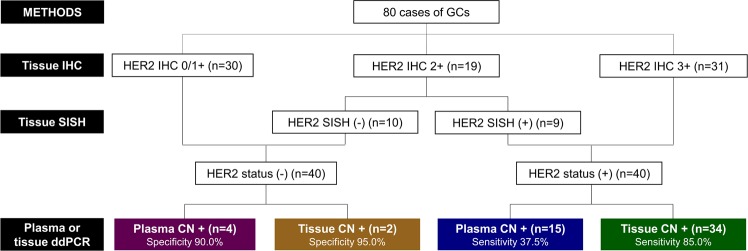
Study profile. GC, Gastric cancer; IHC, Immunohistochemistry; SISH, Silver *in situ* hybridization; CN; Copy number.

Figure 2 summarises the various HER2 results of the enrolled gastric cancer patients in this study. The HER2 status diagnosed with tissue DNA or cfDNA ddPCR methods, the specificity was high (95.0% and 90.0%, respectively), but the sensitivity was low (85.0% and 37.5%, respectively). The concordance rate of plasma cfDNA and tissue DNA is 63.75% (51/80) with 0.235 Cohen’s kappa (Table 2). *HER2* CN of tissue measured by ddPCR is correlated with *HER2* CN of plasma measured by ddPCR (r = 0.232, P = 0.038; Fig. 1d).

**Table 2:** *HER2* copy number status with plasma ddPCR compared to tissue ddPCR.

		Tissue ddPCR		Total
		Negative	Positive	
Plasma ddPCR	Negative	38	23	61
	Positive	6	13	19
Total		44	36	80

ddPCR, droplet digital polymerase chain reaction.

### Correlation between clinicopathological factors and HER2 status

The associations of HER2 status with clinicopathological characteristics of the 80 patients are shown in Table 3. Among the various detection methods, tissue HER2 positive status showed correlation with Lauren classification, especially intestinal type, rather than diffuse type (64.2% vs. 21.7%; P = 0.021), as previously reported^14,15^. The determination of the HER2 status by tissue ddPCR showed significant correlation with aggressive behaviors, including advanced gastric cancer (P = 0.022), presence of lymphatic invasion (P = 0.003), and presence of lymph node metastasis (P = 0.036). However, *HER2* status by plasma *HER2* CN showed no significant association with the features, except HER2 IHC status of tissue samples (P = 0.002, Fig. 1b).

**Table 3:** Clinicopathological features of 80 gastric cancer patients based on various HER2 testing results.

Features	n	Tissue HER2 status by IHC and/or SISH					Tissue *HER2* CN by ddPCR					Plasma *HER2* CN by ddPCR				
		Negative		Positive		*P*	Negative		Positive		*P*	Negative		Positive		*P*
		n	%	n	%		n	%	n	%		n	%	n	%	
Total	80	40	50.0	40	50.0		44	55.0	36	45.0		61	76.3	19	23.8	
Sex																
Female	18	9	50.0	9	50.0	1.000	11	61.1	7	38.9	0.554	15	83.3	3	16.7	0.539^*^
Male	62	31	50.0	31	50.0		33	53.2	29	46.8		46	74.2	16	25.8	
Age (years)																
≤60	27	15	55.6	12	44.4	0.478	16	59.3	11	40.7	0.585	19	70.4	8	29.6	0.378
>60	53	25	47.2	28	52.8		28	52.8	25	47.2		42	79.2	11	20.8	
Depth of invasion																
EGC (pT1)	38	23	60.5	15	39.5	0.073	26	68.4	12	31.6	0.022	32	84.2	6	15.8	0.112
AGC (pT2-4)	42	17	40.5	25	59.5		18	42.9	24	57.1		29	69.0	13	31.0	
Tumour size																
≤3 cm	35	19	54.3	16	45.7	0.499	23	65.7	12	34.3	0.089	28	80.0	7	20.0	0.487
>3 cm	45	21	46.7	24	53.3		21	46.7	24	53.3		33	73.3	12	26.7	
Location																
Lower third	51	26	51.0	25	49.0	0.958	31	60.8	20	39.2	0.334	42	82.4	9	17.6	0.201^**^
Middle third	15	7	46.7	8	53.3		6	40.0	9	60.0		9	60.0	6	40.0	
Upper third	14	7	50.0	7	50.0		7	50.0	7	50.0		10	71.4	4	28.6	
Lauren classification																
Diffuse type	23	18	78.3	5	21.7	0.021^**^	17	73.9	6	26.1	0.135^**^	21	91.3	2	8.7	0.084^**^
Intestinal type	53	19	35.8	34	64.2		24	45.3	29	54.7		37	69.8	16	30.2	
Mixed type	4	3	75.0	1	25.0		3	75.0	1	25.0		3	75.0	1	25.0	
Venous invasion																
Absent	68	34	50.0	34	50.0	1.000	38	55.9	30	44.1	0.706	51	75.0	17	25.0	0.721^*^
Present	12	6	50.0	6	50.0		6	50.0	6	50.0		10	83.3	2	16.7	
Lymphatic invasion																
Absent	37	22	59.5	15	40.5	0.116	27	73.0	10	27.0	0.003	29	78.4	8	21.6	0.678
Present	43	18	41.9	25	58.1		17	39.5	26	60.5		32	74.4	11	25.6	
Perineural invasion																
Absent	49	25	51.0	24	49.0	0.818	30	61.2	19	38.8	0.159	38	77.6	11	22.4	0.731
Present	31	15	48.4	16	51.6		14	45.2	17	54.8		23	74.2	8	25.8	
Lymph node metastasis																
Absent	37	21	56.8	16	43.2	0.262	25	67.6	12	32.4	0.036	29	78.4	8	21.6	0.678
Present	43	19	44.2	24	55.8		19	44.2	24	55.8		32	74.4	11	25.6	
HER2 status by IHC																
0	15	15	100.0	0	0.0	<0.001	15	100.0	0	0.0	<0.001	15	100.0	0	0.0	0.002^**^
1	15	15	100.0	0	0.0		14	93.3	1	6.7		13	86.7	2	13.3	
2	19	10	52.6	9	47.4		10	52.6	9	47.4		14	73.7	5	26.3	
3	31	0	0.0	31	100.0		5	16.1	26	83.9		19	61.3	12	38.7	

*Fisher’s exact test; **Linear by linear association.

EGC, early gastric cancer; AGC, advanced gastric cancer; IHC, immunohistochemistry; SISH, silver *in situ* hybridization; CN, copy number; ddPCR, droplet digital polymerase chain reaction.

**Figure 3:**
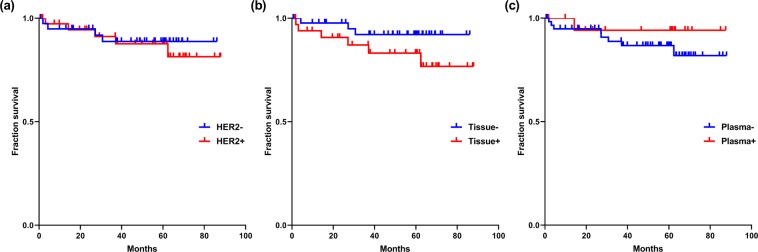
The HER2 positivity and overall survival of the 80 patients. Kaplan-Meier survival curve according to the HER2 positivity assessed by (**a**) IHC and/or SISH (P = 0.700), (**b**) tissue ddPCR (P = 0.146), and (**c**) plasma ddPCR (P = 0.381).

Kaplan-Meier survival analysis was performed to illustrate the prognostic effect of HER2 positivity with various detection methods. The follow-up periods were 1–88 months, with a median follow-up period of 60 months. Among the detection methods, *HER2* positive cases, determined by tissue ddPCR, showed worse outcome than *HER2* negative cases, but without statistical significance (P = 0.146). HER2 positivity by both tissue IHC and/or SISH and plasma ddPCR was not associated with worse prognosis (Fig. 3).

## Discussion

The advanced-stage gastric cancer patients, who are candidates for chemotherapy, should be tested for *HER2* amplification and/or overexpression for trastuzumab chemotherapy^16^. The incidence of intratumoural HER2 heterogeneity is high in gastric cancer, thus larger cancer tissues or multiple biopsy specimens are required for accurate HER2 assessment^17^. However, the small endoscopic biopsy specimen is usually used in HER2 examination for advanced-stage gastric cancer patients because it is difficult to obtain cancer tissues, especially metastatic cancer tissues. Furthermore, serial HER2 assessment for monitoring gastric cancer patients during the follow-up period is not possible with enough cancer tissues. Instead, a blood sample has been suggested to be one of the good candidates to overcome the limitations of cancer tissues. Besides, we had previously demonstrated ddPCR as a useful tool for detecting gene CN in tissue and plasma samples^18^. Therefore, we evaluated the utility and compatibility of ddPCR for *HER2* measurement in gastric cancer, especially using plasma cfDNA.

Previously, several study groups dealt with gastric cancer patients using *HER2* ddPCR. Kinugasa *et al*. included 25 gastric cancer patients and showed that the concordance rate of circulating tumour DNA (ctDNA) and tissue DNA was 62.5%^19^. Also, other groups concluded that tissue *HER2* ddPCR is highly concordant with IHC and/or FISH (95.5% and 100%)^20,21^, which is in line with our results. Two research teams covered *HER2* ddPCR with ctDNA, and one team measured *HER2* ratio using ribonuclease P RNA component H1 (*RPPH1*) as a reference gene and the sensitivity and specificity of plasma *HER2* ddPCR were 73.3% and 93.3%, respectively^22^. The other used elongation factor Tu GTP binding domain containing 2 (*EFTUD2*) as a reference gene and demonstrated the sensitivity and specificity were 76.5% and 83.8%^23^. Compared with the previous studies that measured *HER2* ratios of gastric cancer patients using ddPCR^19–24^, our study covered the largest cohort to the best of our knowledge; moreover, we worked with paired samples of formalin-fixed and paraffin-embedded (FFPE) tissues and preoperative plasma cfDNA. We used *EIF2C1* as the reference gene, and ddPCR using the *HER2/EIF2C1* ratio demonstrated that it can assess HER2 status accurately^25^.

In this study, the specificity of plasma cfDNA *HER2* assessment was high (90.0%), but the sensitivity was low (37.5%). As we demonstrated and discussed in the previous study^18^, ddPCR method for detecting oncogene CN in cfDNA was more specific and less sensitive; our results were concordant with it. The lower sensitivity of our study might be elucidated with the filtration step, which was for the removal of extra components in plasma. There was a chance to lose cfDNA concentration in this step. Also, we used the viral DNA extraction kit which is not intended for cfDNA but suitable for small size DNA. Radical technical advancement in cfDNA analysis including cfDNA collection tubes and extraction kits has been made^26,27^, so there is room for improvement in sensitivity of cfDNA ddPCR with subsequent studies.

According to our study, in advanced gastric cancer, *HER2* positive cases by plasma cfDNA ddPCR may be candidates for trastuzumab treatment, but HER2 assessment by other methods using tissue samples should be done in *HER2* negative cases by ddPCR of plasma cfDNA to overcome low sensitivity. However, the concordance rate between tissue ddPCR and tissue IHC and/or SISH was 90.0% and the sensitivity and specificity of tissue ddPCR were 85.0% and 95.0%, respectively. The false-negative result of tissue ddPCR could be because of the heterogeneity of gastric tumour tissues and a large amount of non-neoplastic cells mixed. Also, it would be because of the difference of measurand, DNA vs. protein. *HER2* gene amplification may not always result in HER2 overexpression^28^. Although the sensitivity was lower than the specificity, tissue ddPCR may replace tissue IHC and/or SISH method. Further studies for the validation of our results are needed.

Previous studies tried to study the clinicopathologic implication of *HER2* amplification, but the relationship between *HER2* amplification and patients’ prognosis has been controversial in gastric cancer^29^. In accordance with the previous studies, plasma *HER2* positivity and tissue HER2 status by IHC and/or SISH did not correlate with aggressive clinicopathologic features or worse prognosis. However, tissue *HER2* positivity determined by ddPCR was significantly correlated with the presence of lymph node metastasis and lymphatic invasion in this study. The patients with tissue *HER2* positive tended to have a worse prognosis, but without statistical significance. It was suggested that *HER2* gene amplification of gastric tissues would result in greater invasive and proliferative tumours^30^, but HER2 status itself is not a strong prognostic biomarker by any detection method.

The limitation of our study was that it was a cross-sectional and retrospective study. We retrospectively collected the preoperative blood and tissue samples of gastric cancer patients who were treated by surgeries. Further prospective studies with larger cohorts will be needed. In addition, HER2 measurement in patients after gastrectomy and during the follow-up period will give more information about relapse or treatment effects of trastuzumab^23,31^. The other limitation is that there has been no consensus about setting cutoffs for *HER2* amplification. For *HER2* CN analysis, it is required to quantify the ratio of a target sequence to a reference, and we used 2.510 as a cutoff for *HER2* amplification of plasma cfDNA. Meanwhile, we set the cutoff of tissue *HER2* amplification with a ROC curve. However, Kinugasa *et al*. set the *HER2* cutoff value as 1.2 using the healthy serum and tissue samples which corresponds to 2.4 for CN^19^, and there was no difference between the analyses whether the cutoff value is different for plasma and tissue or not. In other studies^22,24^, they set the cutoff values of 2.1 and 2.13, which is slightly lower than our cutoff value that was measured with the healthy volunteers’ plasma. So, the consensus for cutoff of *HER2* CN will be needed.

In conclusion, *HER2* ddPCR using tissue and plasma samples was able to detect HER2 amplification and/or overexpression in patients with gastric cancers. *HER2* CN testing by ddPCR had high specificity and low sensitivity; thus, tissue HER2 IHC/SISH examination would be necessary when *HER2* ddPCR shows negative results. In addition, *HER2* ddPCR testing in tissue and plasma samples could supplement or substitute the HER2 testing of gastric cancers, especially if it is difficult to obtain enough tissue samples in clinical practice.

## Methods

### Patients and samples

The subjects of our study were 80 patients who were diagnosed with gastric adenocarcinoma and underwent surgery at the Seoul National University Bundang Hospital between 2011 and 2012. The patients had undergone radical gastrectomy with D2 lymph node dissection, followed by conventional adjuvant or palliative chemotherapy if clinically indicated or TNM stage was 2 or more. Considering the low incidence of HER2 positivity in gastric cancer patients, 31 cases of HER2 IHC 3+, 19 cases of IHC 2+ cases, and 30 cases of IHC 0 or 1+ cases were included in this study. Clinicopathological information of patients was collected retrospectively from the electronic medical records, including patient outcome and overall survival. The stages of cancer were determined according to the 8^th^ edition of the American Joint Committee on Cancer (AJCC)^32^. Preoperative blood samples were taken 1–20 days before the operation. The formalin-fixed paraffin-embedded (FFPE) gastric normal and tumour tissues were also collected after surgery. In addition, to set a cutoff value of *HER2* CN for plasma, we recruited 15 healthy volunteers who have no underlying diseases, including cancer, and collected their blood samples by venous puncture. None of them working at our hospital was included in this study, and the study population including healthy control participated in our study voluntarily. This study was performed with the written informed consent of all subjects and approval of the Institutional Review Board of SNUBH (IRB No. B-1005/099-009). This study was done under the observance of the Bioethics Law of South Korea.

### HER2 immunohistochemistry and silver *in situ* hybridization

Immunohistochemical staining was done by an automatic immunostainer (BenchMark XT, Ventana Medical Systems, Inc., Tucson, AZ, USA) using an anti-HER2/neu antibody (4B5; pre-dilution; Ventana Medical Systems) according to the manufacturer’s guideline. Bright-field dual-color SISH was performed with the automatic SISH stainer (BenchMark XT, Ventana Medical Systems) using INFORM *HER2* DNA and INFORM Chromosome 17 (CEP17) probes (Ventana Medical Systems). HER2 status of the FFPE tissues was evaluated with IHC and SISH by an expert pathologist (H.S.L.), as described in the previous study^33^. According to the DAKO guidelines for scoring HercepTest™ in gastric cancer, IHC scores of 0 and 1+ were considered as HER2 negative, whereas IHC 3+ was considered as HER2 positive. If IHC score is 2+ and SISH score >2.0, it was regarded as HER2 positive^34^.

### DNA preparation from tissue samples

The FFPE tissue samples of the 80 patients were deparaffinised by boiling at 70 °C for 10 min and centrifuged for 10 min at 13000 rpm. Tissues were dissected to four 8 μm-thick sections, which included sufficient tumour cells confirmed with H&E-stained slides, and the represented area contained 60% or more tumour cells. DNA was extracted from each FFPE sample by QIAamp DNA FFPE Tissue Kit (Qiagen, Hilden, Germany). The DNA concentration was determined by a NanoDrop 2000 spectrophotometer (Thermo Scientific, Waltham, MA, USA) and DNA samples >50 ng/μL were diluted into 50 ng/μL.

### Circulating DNA isolation from blood samples

Blood samples from 80 patients (before surgery) and 15 healthy people were obtained in EDTA tubes, and plasma was separated from the cellular fraction within 2 h of collection by centrifuging at 3000 rpm for 10 min at room temperature. Before the polymerase chain reaction, plasma was filtered with Fisherbrand™ Standard Serum Filters (Fisher Scientific, Waltham, MA, USA) and DNA was extracted by High Pure Viral Nucleic Acid Kit (Roche, Basel, Switzerland) from 300 μL of filtered plasma.

### Digital droplet PCR for *HER2* copy number analysis

Digital droplet PCR was done to measure *HER2* CN from the DNA extracted from tissues and plasma using QX200 Droplet Digital PCR System (Bio-Rad, Hercules, CA, USA) according to the manufacturer’s protocols. The PCR was performed with the C1000 Touch™ Thermal Cycler (Bio-Rad) at 95 °C for 10 min, 94 °C for 30 sec with 50 cycles, 60 °C for 1 min, and 98 °C for 10 min. The target gene erb-b2 receptor tyrosine kinase 2 (*ERBB2*) was labeled with FAM, and the reference gene *EIF2C1* was labeled with HEX. Data analysis was performed with the QuantaSoft software (version 1.7.4.; Bio-Rad). The *HER2/EIF2C2* ratio was defined as *HER2* ratio, and the CN was calculated by multiplying the ratio by 2.

### Statistical analysis

For the categorical variables, Chi-square test, Fisher’s exact test or linear by linear association test were used as appropriate. Continuous variables were compared using Student’s t-test, Mann-Whitney U test or Jonckheere-Terpstra test, according to the data types. Survival analysis was calculated using the Kaplan-Meier curves, and statistical significance was analyzed by the log-rank test. All statistical tests were two-tailed and performed with SPSS version 25.0. (IBM, Armonk, NY, USA). Statistical significance was considered when P-values were <0.05.

## Supplementary information

Supplementary Figure S1.

## References

[1] International Agency for Research on Cancer. Global Cancer Observatory http://gco.iarc.fr/ (2018).

[2] Fornaro L (2011). Anti-HER agents in gastric cancer: from bench to bedside. Nat. Rev. Gastroenterol. Hepatol..

[3] Gravalos C, Jimeno A (2008). HER2 in gastric cancer: a new prognostic factor and a novel therapeutic target. Ann. Oncol..

[4] Hudis CA (2007). Trastuzumab–mechanism of action and use in clinical practice. N. Engl. J. Med..

[5] Bang YJ (2010). Trastuzumab in combination with chemotherapy versus chemotherapy alone for treatment of HER2-positive advanced gastric or gastro-oesophageal junction cancer (ToGA): a phase 3, open-label, randomised controlled trial. Lancet.

[6] Ruschoff J (2012). HER2 testing in gastric cancer: a practical approach. Mod. Pathol..

[7] National Comprehensive Cancer Network. *NCCN Clinical Practice Guidelines in Oncology (NCCN Guidelines) Gatric Cancer (Version 2.2018)* https://www.nccn.org/professionals/physician_gls/pdf/gastric.pdf (2018).

[8] Bartley AN (2017). HER2 Testing and Clinical Decision Making in Gastroesophageal Adenocarcinoma: Guideline From the College of American Pathologists, American Society for Clinical Pathology, and the American Society of Clinical Oncology. J. Clin. Oncol..

[9] Wong N (2018). HER2 testing of gastro-oesophageal adenocarcinoma: a commentary and guidance document from the Association of Clinical Pathologists Molecular Pathology and Diagnostics Committee. J. Clin. Pathol..

[10] Shah MA, Kelsen DP (2010). Gastric cancer: a primer on the epidemiology and biology of the disease and an overview of the medical management of advanced disease. J. Natl Compr. Canc Netw..

[11] Nishida Y (2015). A novel gene–protein assay for evaluating HER2 status in gastric cancer: simultaneous analyses of HER2 protein overexpression and gene amplification reveal intratumoral heterogeneity. Gastric Cancer.

[12] Wan JCM (2017). Liquid biopsies come of age: towards implementation of circulating tumour DNA. Nat. Rev. Cancer.

[13] Hudecova I (2015). Digital PCR analysis of circulating nucleic acids. Clin. Biochem..

[14] Im SA (2011). Clinicopathologic characteristics of patients with stage III/IV (M(0)) advanced gastric cancer, according to HER2 status assessed by immunohistochemistry and fluorescence *in situ* hybridization. Diagn. Mol. Pathol..

[15] Baretton G (2019). HER2 testing in gastric cancer diagnosis: insights on variables influencing HER2-positivity from a large, multicenter, observational study in Germany. Virchows Arch..

[16] Lee HS (2017). Molecular Testing for Gastrointestinal Cancer. J. Pathol. Transl. Med..

[17] Lee HE (2013). Clinical significance of intratumoral HER2 heterogeneity in gastric cancer. Eur. J. Cancer.

[18] Lee KS (2019). Digital polymerase chain reaction for detecting c-MYC copy number gain in tissue and cell-free plasma samples of colorectal cancer patients. Sci. Rep..

[19] Kinugasa H (2015). Droplet digital PCR measurement of HER2 in patients with gastric cancer. Br. J. Cancer.

[20] Zhu Y (2016). Droplet digital polymerase chain reaction detection of HER2 amplification in formalin fixed paraffin embedded breast and gastric carcinoma samples. Exp. Mol. Pathol..

[21] Wang X (2017). Human epidermal growth factor receptor 2 amplification detection by droplet digital polymerase chain reaction in formalin-fixed paraffin-embedded breast and gastric cancer samples. J. Cancer Res. Ther..

[22] Shoda K (2017). Monitoring the HER2 copy number status in circulating tumor DNA by droplet digital PCR in patients with gastric cancer. Gastric Cancer.

[23] Liu Y (2019). Quantitative Analysis of HER2 Amplification by Droplet Digital PCR in the Follow-Up of Gastric Cancer Patients Being Treated with Trastuzumab after Surgery. Gastroenterol. Res. Pract..

[24] Shoda K (2015). HER2 amplification detected in the circulating DNA of patients with gastric cancer: a retrospective pilot study. Gastric Cancer.

[25] Tantiwetrueangdet A, Panvichian R, Wongwaisayawan S, Sueangoen N, Lertsithichai P (2018). Droplet digital PCR using HER2/EIF2C1 ratio for detection of HER2 amplification in breast cancer tissues. Med. Oncol..

[26] van Dessel LF (2017). Application of circulating tumor DNA in prospective clinical oncology trials – standardization of preanalytical conditions. Mol. Oncol..

[27] Sorber L (2017). A Comparison of Cell-Free DNA Isolation Kits: Isolation and Quantification of Cell-Free DNA in Plasma. J. Mol. Diagnostics.

[28] Meric-Bernstam F (2019). Advances in HER2-Targeted Therapy: Novel Agents and Opportunities Beyond Breast and Gastric Cancer. Clin. Cancer Res..

[29] Boku N (2014). HER2-positive gastric cancer. Gastric Cancer.

[30] Wang HB, Liao XF, Zhang J (2017). Clinicopathological factors associated with HER2-positive gastric cancer: A meta-analysis. Med..

[31] Wang D-S (2019). Liquid biopsies to track trastuzumab resistance in metastatic HER2-positive gastric cancer. Gut.

[32] Amin, M.B. *AJCC cancer staging manual* (Springer, Chicago (2017).

[33] Seo AN (2014). HER2 status in colorectal cancer: its clinical significance and the relationship between HER2 gene amplification and expression. PLoS One.

[34] Hofmann M (2008). Assessment of a HER2 scoring system for gastric cancer: results from a validation study. Histopathology.

