# Author Correction: Comparative analysis of *HER2* copy number between plasma and tissue samples in gastric cancer using droplet digital PCR

**DOI:** 10.1038/s41598-020-65179-7

**Published:** 2020-05-15

**Authors:** Boram Kim, Soo Kyung Nam, Soo Hyun Seo, Kyoung Un Park, Sang-Hoon Ahn, Do Joong Park, Hyung-Ho Kim, Woo Ho Kim, Hye Seung Lee

**Affiliations:** 1 Department of Laboratory Medicine, Seoul National University College of Medicine, Seoul, 03080 Republic of Korea; 2 Department of Laboratory Medicine, Seoul National University Hospital, Seoul, 03080 Republic of Korea; 3 Department of Pathology, Seoul National University Bundang Hospital, Seongnam, 13620 Republic of Korea; 4 Department of Laboratory Medicine, Seoul National University Bundang Hospital, Seongnam, 13620 Republic of Korea; 5 Department of Surgery, Seoul National University College of Medicine, Seoul, 03080 Republic of Korea; 6 Department of Surgery, Seoul National University Bundang Hospital, Seongnam, 13620 Republic of Korea; 7 Department of Pathology, Seoul National University College of Medicine, Seoul, 03080 Republic of Korea

Correction to: *Scientific Reports* 10.1038/s41598-020-60897-4, published online 06 March 2020

In the HTML and PDF of the original version of this Article Hye Seung Lee was incorrectly affiliated with “Department of Laboratory Medicine, Seoul National University Hospital, Seoul, 03080, Republic of Korea” The correct affiliations for Hye Seung Lee are listed below.

Department of Pathology, Seoul National University Bundang Hospital, Seongnam, 13620, Republic of Korea.

Department of Pathology, Seoul National University College of Medicine, Seoul, 03080, Republic of Korea.

Additionally, the Supplementary Figure S1 file contained errors in the affiliation list where affiliations 2, 4 and 6 were omitted. As a result, affiliation 3 was listed as affiliation 2, affiliation 5 was listed as affiliation 3 and affiliation 7 was listed as affiliation 4.

These errors have now been corrected in the PDF and HTML versions of the Article, and in the accompanying Supplementary Information file.

