# Supplementary material for: Comparative analysis of HER2 copy number between plasma and tissue samples in gastric cancer using droplet digital PCR
Source: Sci Rep. 2020 Mar 6;10:4177. doi: 10.1038/s41598-020-60897-4 (PMC7060190; doi:10.1038/s41598-020-60897-4)

Comparative analysis of *HER2* copy number between plasma and tissue samples in gastric cancer using droplet digital PCR

Boram Kim[1,2], Soo Kyung Nam[3], Soo Hyun Seo[1,4], Kyoung Un Park[1,4], Sang-Hoon Ahn[5,6], Do Joong Park[5], Hyung-Ho Kim[5,6], Woo Ho Kim[7], Hye Seung Lee[3,7*]

[1]Department of Laboratory Medicine, Seoul National University College of Medicine, Seoul, 03080, Republic of Korea

[2]Department of Laboratory Medicine, Seoul National University Hospital, Seoul, 03080, Republic of Korea

[3]Department of Pathology, Seoul National University Bundang Hospital, Seongnam, 13620, Republic of Korea

[4]Department of Laboratory Medicine, Seoul National University Bundang Hospital, Seongnam, 13620, Republic of Korea

[5]Department of Surgery, Seoul National University College of Medicine, Seoul, 03080, Republic of Korea

[6]Department of Surgery, Seoul National University Bundang Hospital, Seongnam, 13620, Republic of Korea

[7]Department of Pathology, Seoul National University College of Medicine, Seoul, 03080, Republic of Korea

*** Corresponding author:** Hye Seung Lee, MD, PhD

E-mail address: hye2@snu.ac.kr

Supplementary Figure S1. Receiver Operating Characteristics curve of tissue *HER2* ddPCR, area under the curve (AUC) 0.963 (95% CI, 0.928-0.998; P<0.001).

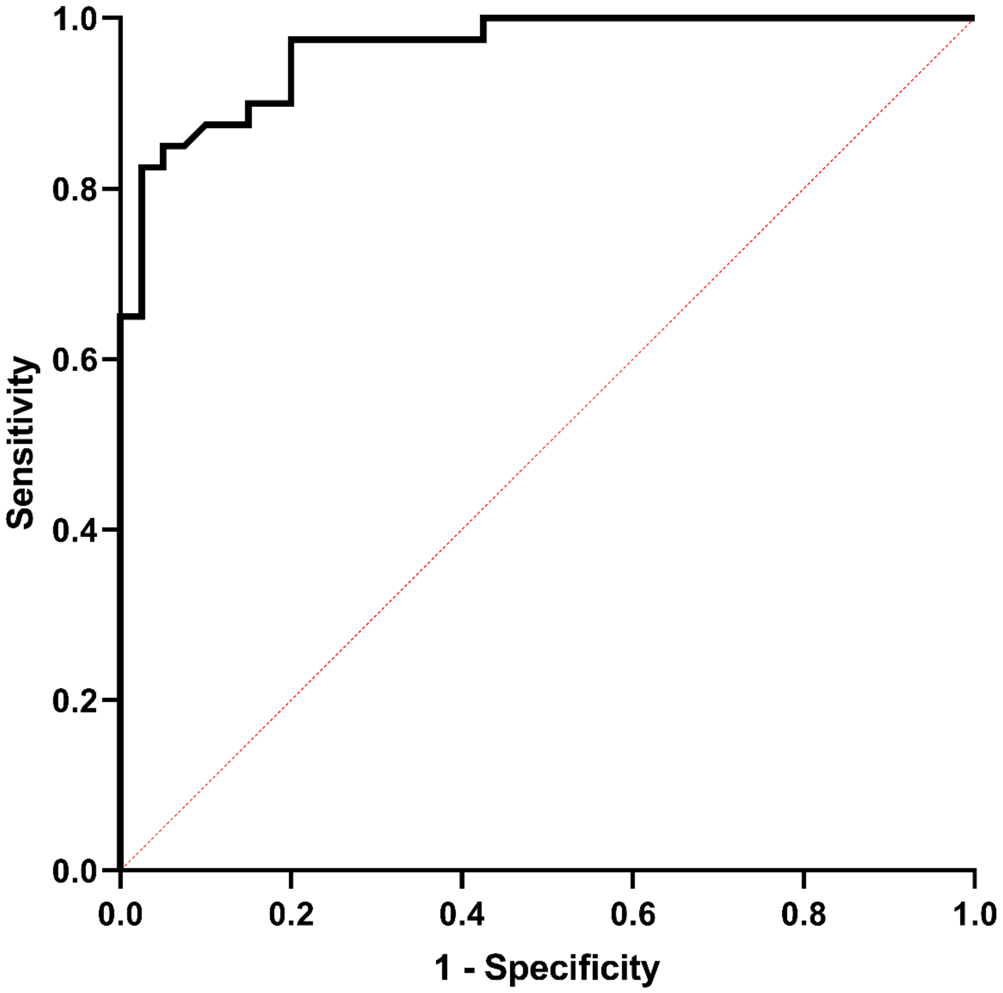

Supplement: Supplementary file 1 — Supplementary Figure S1. [file 41598_2020_60897_MOESM1_ESM.pdf]
